# Retinal and Choroidal Microvascular Profiles in Professional Handball Players: A Cross-Sectional OCTA Study

**DOI:** 10.3390/vision10040077

**Published:** 2026-10-06

**Authors:** Hakika Erdoğan, Melike Taşkan, Çağlar Bektaş, Mustafa Deniz Dindar, Gülçin Usta

**Affiliations:** 1 Department of Ophthalmology, Faculty of Medicine, Çanakkale Onsekiz Mart University, Barbaros Mahallesi, Sevim Buluç Caddesi, 17100 Çanakkale, Turkey; 2 Faculty of Sport Sciences, Çanakkale Onsekiz Mart University, Barbaros Mahallesi, Sevim Buluç Caddesi, 17100 Çanakkale, Turkey

**Keywords:** optical coherence tomography angiography, professional athletes, handball, retinal microvasculature, choroidal microvasculature, exercise

## Abstract

The long-term association between professional athletic training and ocular microvascular characteristics remains unclear. This cross-sectional study compared retinal and choroidal optical coherence tomography angiography (OCTA) parameters between 31 male professional handball players with at least five years of training and 30 age-matched sedentary male controls. The right eye of each participant was imaged using a 6 × 6 mm OCTA macular scan (NIDEK RS-3000 Advance). Foveal avascular zone (FAZ) metrics, vessel density (VD), perfusion density (PD), retinal nerve fiber layer (RNFL) thickness, and ganglion cell complex (GCC) thickness were evaluated, and *p* values were adjusted using the Benjamini–Hochberg false discovery rate procedure. FAZ metrics, RNFL thickness, and GCC thickness did not differ significantly between groups after correction for multiple comparisons. In contrast, all eight retinal and choroidal VD and PD parameters were lower in professional handball players, with adjusted *p* values ranging from <0.001 to 0.004 and moderate-to-large effect sizes (rank-biserial r = 0.449–0.668). Professional handball participation was associated with lower OCTA-derived retinal and choroidal vascular metrics despite comparable structural OCT parameters. Longitudinal studies are needed to confirm the physiological significance and causal basis of this pattern.

## 1. Introduction

The human retina offers a unique opportunity to study systemic microvascular physiology because it is the only vascular bed that can be directly visualized non-invasively in vivo. Together with the choroid, it provides an accessible reflection of systemic vascular health. Unlike the retinal circulation, which is tightly autoregulated and largely independent of autonomic control, the choroidal circulation is richly innervated, only weakly autoregulated, and closely mirrors systemic haemodynamics [1,2]. Consequently, optical coherence tomography angiography (OCTA)-derived retinal and choroidal microvascular parameters have emerged as promising biomarkers of systemic vascular health and may provide prognostic information in cardiovascular and metabolic diseases [3].

Regular exercise is one of the most potent physiological stimuli influencing the cardiovascular system and microcirculation. Beyond the transient vasomotor responses elicited by a single bout of exercise, sustained training induces durable structural and functional adaptations, including improved endothelial function, nitric oxide-mediated vasodilation, vascular remodelling, and shifts in autonomic balance, all of which are thought to contribute substantially to the cardiovascular benefits of regular physical activity [3,4]. Understanding these long-term physiological adaptations is important not only for optimizing athletic performance but also for improving our understanding of human vascular physiology. However, how the retinal and choroidal microvasculature ultimately adapts to years of sustained exercise remains incompletely understood.

OCTA enables rapid, depth-resolved, quantitative, and repeatable assessment of retinal and choroidal microvascular architecture without the need for intravenous contrast, making it particularly suitable for longitudinal physiological studies and repeated monitoring of exercise-induced vascular adaptation. Existing OCTA studies have predominantly investigated acute exercise or short-term training interventions, and even these findings remain inconsistent. Acute exercise has been shown to transiently reduce retinal vessel density (VD) in proportion to systemic blood pressure changes, followed by rapid recovery and a characteristic retina–choroid dissociation [1,2,5,6,7,8]. In contrast, studies evaluating resistance or interval training have reported reduced, increased, or unchanged retinal microvascular parameters depending on exercise modality, participant characteristics, and imaging methodology [4,9,10,11,12]. Furthermore, greater aerobic fitness has only been cross-sectionally associated with a smaller foveal avascular zone (FAZ) [13]. Importantly, most published studies have included recreationally active individuals, older adults, or clinical populations [4,11,14,15], whereas the retinal and choroidal microvasculature of athletes exposed to many years of continuous professional training has rarely been investigated [12,16].

The ocular microvascular characteristics of athletes exposed to long-term professional handball participation remain insufficiently understood. Professional handball players represent a relatively homogeneous athletic population exposed to years of combined resistance, aerobic, and high-intensity intermittent activity. Comparison with age- and sex-matched healthy non-athletic controls may therefore provide insight into the retinal and choroidal microvascular profile associated with professional handball participation. However, because cross-sectional comparisons cannot establish temporal or causal relationships, any observed differences should be interpreted as associations rather than direct consequences of long-term training.

Using OCTA, this study compared retinal and choroidal microvascular parameters between professional male handball players with at least five years of continuous participation and age-matched healthy male non-athletic controls. We aimed to determine whether professional handball players exhibit a distinct ocular microvascular profile and to assess whether any vascular differences are accompanied by alterations in FAZ metrics, retinal nerve fiber layer (RNFL) thickness, or ganglion cell complex (GCC) thickness.

## 2. Materials and Methods

### 2.1. Study Design and Participants

This cross-sectional, single-center study was conducted between 25 July and 15 August 2026 at the Department of Ophthalmology, Çanakkale Onsekiz Mart University Faculty of Medicine. Professional handball players who attended routine ophthalmic screening examinations during the pre-season preparation period were consecutively recruited. To minimize the influence of diurnal variation on retinal and choroidal perfusion, all ophthalmic examinations and OCTA acquisitions were performed during the same predefined time period under standardized examination conditions.

The study included 31 professional male handball players and 30 healthy male non-athletic controls (Table 1). The mean age of the handball players was 23.42 ± 3.24 years, compared with 23.63 ± 1.52 years in the control group, with no significant difference between groups (*p* = 0.941). Ages ranged from 16 to 32 years among athletes and from 21 to 27 years among controls.

The study adhered to the tenets of the Declaration of Helsinki and was approved by the Ethics Committee of Çanakkale Onsekiz Mart University (protocol code: 2026-11/11-24; date of approval: 22 July 2026). Written informed consent was obtained from adult participants prior to enrollment. For the three athletes younger than 18 years, written informed consent was obtained from a parent or legal guardian, together with the participant’s assent.

### 2.2. Training Characteristics

Professional athletes were recruited from the same handball club competing in the same national league. All athletes had participated in professional handball for at least five consecutive years and followed a relatively homogeneous training program consisting of approximately 3 h of supervised training every other day. All examinations were performed during the pre-season preparation period while athletes maintained their regular training schedule. The last training session took place one day before OCTA imaging. Because the athletes arrived together from the same training camp, their meal and rest periods before examination were standardized. All athletes belonged to the same professional team and followed the same coaching staff and training schedule throughout the pre-season preparation period, thereby minimizing inter-individual variability related to training exposure.

The control group consisted of healthy age- and sex-matched volunteers who did not participate in competitive sports or structured exercise programs. None attended a fitness center or engaged in regular endurance training, resistance training, or planned walking programs, and all were classified as sedentary based on their habitual physical activity.

### 2.3. Inclusion and Exclusion Criteria

Only participants with a best-corrected visual acuity (BCVA) of 20/20 (Snellen equivalent), a spherical equivalent between −0.50 and +0.50 diopters, and an intraocular pressure (IOP) between 10 and 21 mmHg were included.

All participants underwent a comprehensive ophthalmic examination including BCVA assessment using a Snellen chart, objective refraction using a TONOREF III auto ref/kerato/tono/pachymeter (NIDEK Co., Ltd., Gamagori, Aichi, Japan), slit-lamp biomicroscopy using an SL 9900 slit lamp (CSO, Florence, Italy), IOP measurement using a Goldmann applanation tonometer (L-5110, Inami & Co., Ltd., Tokyo, Japan), and dilated fundus examination using a 90-diopter non-contact lens (Volk Optical Inc., Mentor, OH, USA).

Participants were excluded if they had any history of:

- ocular surgery;
- ocular trauma;
- retinal disease;
- corneal disease;
- glaucoma;
- optic nerve disease;
- systemic diseases known to affect the ocular microvasculature (including diabetes mellitus and hypertension);
- smoking;
- alcohol consumption;
- regular medication use;
- or any condition preventing acquisition of high-quality OCTA images.

Eyes with media opacity, unstable fixation, poor image quality, or inadequate OCTA signal strength were also excluded.

To avoid inter-eye correlation, only the right eye of each participant was included in the statistical analysis.

### 2.4. Optical Coherence Tomography Angiography

OCTA imaging was performed using the RS-3000 Advance system (NIDEK Co., Ltd., Gamagori, Aichi, Japan; NAVIS-EX software version 1.10.0.11). All scans were obtained under standardized conditions using the same device. All OCTA examinations were performed between 09:00 and 11:00 a.m. to minimize the influence of diurnal variation on retinal and choroidal perfusion. Only images with a signal strength index (SSI) of ≥6 were included in the analysis. All images were reviewed for segmentation errors, motion artifacts, blink-related signal loss, vessel duplication, and poor fixation. Images not meeting these quality criteria were excluded and repeated whenever possible. Automated segmentation boundaries were checked before quantitative analysis.

Macular scans were acquired using a 6 × 6 mm scanning protocol centered on the fovea. Measurements were evaluated according to the Early Treatment Diabetic Retinopathy Study (ETDRS) grid. The inner ring (1–3 mm) represented the parafoveal region, whereas the outer ring (3–6 mm) represented the perifoveal region. For each parameter, the mean of the inner- and outer-ring measurements was used in the analysis.

The retinal layers were analyzed using the device’s automated segmentation algorithm and the AngioScan OCTA analysis functions in NAVIS-EX. The recorded parameters included FAZ area, perimeter, and circularity; VD and perfusion density (PD) of the superficial capillary plexus (SCP), deep capillary plexus (DCP), choriocapillaris (CC), and choroid; and optic nerve head (ONH), radial peripapillary capillary plexus (RPC), and lamina cribrosa (LC) measurements. The software automatically calculates VD as the total length of detected perfused vessels per unit area (mm/mm^2^), irrespective of vessel width, and PD as the percentage of the analyzed area occupied by detected perfused vessels, which incorporates vessel width [17]. Thus, VD and PD capture complementary aspects of the OCTA signal. Individual vessel counts and vessel calibers were not measured separately.

Structural optical coherence tomography (OCT) measurements of RNFL thickness and GCC thickness were obtained using the same imaging system. RNFL thickness was measured using the optic disc scanning protocol, whereas GCC thickness was obtained using the macular analysis protocol provided by the manufacturer. All images were reviewed by the same experienced ophthalmologist before inclusion in the final analysis.

### 2.5. Statistical Analysis

Statistical analyses were performed using Python (version 3.12.3) with the SciPy library (version 1.17.1).

Continuous variables are presented as mean ± standard deviation (SD) and, in Tables 2 and 3, also as median (interquartile range). All statistical tests were two-tailed.

The distribution of continuous variables was assessed separately in each study group using the Shapiro–Wilk test. Homogeneity of variances was evaluated using Levene’s test.

Variables satisfying both the normality and homogeneity assumptions were compared using the independent-samples *t*-test. Variables violating the normality assumption in either group were analyzed using the Mann–Whitney U test. Only RNFL thickness fulfilled all parametric assumptions and was therefore analyzed using the independent-samples *t*-test, whereas all remaining OCTA parameters were compared using the Mann–Whitney U test.

Because tied values were present in the OCTA variables, the asymptotic Mann–Whitney U test with tie correction was applied.

Effect sizes were reported as Cohen’s d for independent-samples *t*-tests and as the rank-biserial correlation (r) for Mann–Whitney U tests. For the only parametric comparison, RNFL thickness, the observed Cohen’s d was −0.40 (Table 2).

Because no a priori sample-size calculation was performed, we conducted a sensitivity calculation for the enrolled sample of 31 athletes and 30 controls. An assumed d = 0.75 was used as an illustrative moderate-to-large standardized difference in the absence of prior data for professional handball players. Under a two-sided independent-group comparison with α = 0.05, an assumed standardized mean difference (Cohen’s d) of 0.75 requires approximately 29 participants per group for 80% power; the enrolled sample provides approximately 82% power for that assumed effect. Conversely, with 31 and 30 participants, the standardized difference corresponding to 80% power is approximately d = 0.73. These calculations use a two-sample *t*-test approximation, do not account for false discovery rate (FDR) adjustment, and should not be interpreted as an a priori design calculation or as power estimates for the rank-based primary comparisons.

To control for multiple comparisons across the 13 reported OCTA and structural outcomes, raw *p* values were adjusted together using the Benjamini–Hochberg FDR procedure. Demographic variables were analyzed separately and were not included in this correction. A two-sided FDR-adjusted *p* value < 0.05 was considered statistically significant.

For each outcome, only participants with an available measurement were included in that comparison; missing outcome measurements were not imputed. Accordingly, the analyzed sample sizes varied: RNFL thickness, 30 athletes and 28 controls; GCC thickness, 31 and 18; DCP VD, 28 and 29; DCP PD, 29 and 30. The other outcomes included all 31 athletes and 30 controls (Tables 2 and 3).

During manuscript preparation and revision, the authors used ChatGPT GPT-5 (OpenAI) for assistance with statistical coding and Claude Opus 5.5 (Anthropic) for an editorial and consistency review of the revisions. All calculations, text, and outputs were reviewed and verified by the authors, who take full responsibility for the analyses and content.

## 3. Results

A total of 61 participants were included in the final analysis, comprising 31 professional handball players and 30 healthy non-athletic controls. The demographic characteristics of the study population are presented in Table 1. The two groups were comparable in age, with no statistically significant difference observed (*p* = 0.941), indicating successful demographic matching between professional athletes and controls. 

BCVA was 1.0 (decimal notation) in both groups; participants with reduced visual acuity were excluded. Mean Goldmann applanation tonometry measurements were 12.3 ± 2.7 mmHg in handball players and 12.5 ± 2.0 mmHg in controls (Table 1). Refractive error was restricted to a spherical equivalent between −0.50 and +0.50 D by the eligibility criteria. These examination findings are descriptive; no additional between-group statistical comparisons were performed.

The structural OCT and OCTA findings are summarized in Table 2. After adjustment for multiple comparisons using the Benjamini–Hochberg FDR procedure, none of the structural parameters differed significantly between professional handball players and non-athletic controls. Specifically, FAZ area, FAZ perimeter, RNFL thickness, and GCC thickness were comparable between groups. Although FAZ circularity demonstrated a nominally significant raw *p* value, this association did not remain significant after FDR correction. 

In contrast, retinal and choroidal microvascular OCTA parameters demonstrated a highly consistent pattern of between-group differences (Table 3). All retinal and choroidal VD and PD parameters were significantly lower in professional handball players than in non-athletic controls, and all remained statistically significant after adjustment for multiple comparisons. This pattern was observed across every vascular layer examined, including the SCP, DCP, CC, and choroid. 

Among all OCTA parameters, SCP VD demonstrated the largest between-group effect size (r = 0.668), followed by SCP PD (r = 0.626) and CC PD (r = 0.574). The remaining microvascular parameters—including DCP VD, DCP PD, choroidal VD, choroidal PD, and CC VD—also demonstrated statistically significant differences with moderate-to-large effect sizes. Importantly, every significant parameter showed the same directional pattern, with lower VD and PD values observed in professional handball players relative to non-athletic controls.

Across the evaluated vascular layers, OCTA-derived VD and PD were lower in professional handball players than in controls, while the measured structural OCT and FAZ parameters did not differ significantly after FDR adjustment. The clinical and physiological significance of these associations remains uncertain.

## 4. Discussion

Professional handball players with at least five years of training had lower OCTA-derived VD and PD than age- and sex-matched sedentary controls in all eight retinal and choroidal comparisons, with moderate-to-large effect sizes (r = 0.449–0.668). The difference had the same direction in every layer examined (SCP, DCP, CC, and choroid) and persisted after FDR correction, whereas FAZ metrics, RNFL thickness, and GCC thickness did not differ significantly after correction. Because the vascular difference was consistent across layers rather than confined to a single plexus, it is less likely to reflect a segmentation error in one slab. However, the cross-sectional design establishes an association only, and OCTA does not measure absolute blood flow or oxygen delivery.

Several mechanisms could contribute to this pattern. One hypothesis is enhanced microvascular efficiency: in older adults, reduced retinal capillary density after 24 weeks of high-speed circuit training was interpreted as more efficient use of the perfused capillary network rather than impaired perfusion [9]. Retinal autoregulation and exercise-related improvements in endothelial function and nitric oxide bioavailability might also influence OCTA-derived density without structural change [13], and choroidal perfusion has been reported to remain within a stable range after 8 and 24 weeks of circuit training [10,11]. These explanations remain speculative. VD reflects the total length of detected perfused vessels per unit area, whereas PD reflects the proportion of the area occupied by detected perfused vessels and therefore also depends on vessel width [17]. Neither metric distinguishes fewer perfused capillaries from narrower or less detectable ones, and neither measures flow velocity, volumetric flow, or oxygenation. Lower values could therefore reflect physiological adaptation, reduced capillary recruitment at rest, or other factors, and the present data cannot discriminate between these possibilities.

The absence of significant differences in RNFL thickness, GCC thickness, and FAZ geometry suggests that the vascular differences were not accompanied by detectable neuroretinal structural change. This dissociation is plausible in healthy young adults: RNFL and GCC mainly reflect neuronal and axonal integrity, whereas VD and PD describe the perfused microvasculature, which may respond more readily to haemodynamic and autoregulatory influences. Similar dissociations have been reported. Aerobic fitness has been associated with FAZ morphology without parallel changes in retinal flow density [13]; acute intensive exercise produces transient retinal and choroidal thickness changes that resolve rapidly [15]; isometric and dynamic exercise alter optic nerve head and macular perfusion while largely preserving morphology [2,8]; and endurance exercise has been associated with divergent changes in retinal thickness and vascular density [7]. Nevertheless, non-significant structural comparisons do not exclude smaller effects. With the present sample, only standardized differences of approximately d = 0.73 or larger could be detected with 80% power, and GCC measurements were available for only 18 controls.

Our findings are only partly consistent with previous training studies. In healthy older adults, retinal VD decreased after 24 weeks of high-speed circuit training, whereas CC density remained unchanged [9,10,11]. In young football players, a single Wingate test produced no significant change in retinal or choroidal VD apart from a modest reduction in DCP FAZ area [16], whereas six weeks of high-intensity interval training increased DCP and CC VD [12]. Four weeks of interval training in individuals with type 1 diabetes produced no significant OCTA change despite improved fitness [4], and moderate exercise in children produced variable, layer-specific responses [14]. These studies differ from ours in participant age and health status, exercise modality, duration of exposure, and the interval between exercise and imaging. They also differ in OCTA devices, segmentation algorithms, and density definitions, which substantially affect quantitative values [5,6]. The discrepancies therefore cannot be attributed to training duration alone. Our cohort, exposed to at least five years of professional training, extends these observations to a population that has rarely been studied.

The retinal and choroidal circulations are regulated differently. The retinal circulation is tightly autoregulated and largely independent of autonomic input, whereas the choroid is richly innervated, weakly autoregulated, and more closely reflects systemic haemodynamics [3]. Acute exercise studies accordingly report divergent responses, such as increased retinal but decreased choroidal thickness after a marathon [7] and a transient retina–choroid dissociation during early recovery [15]. In contrast, our athletes showed lower values in both circulations. Imaging on the day after training, rather than immediately after exercise, may partly explain why the transient haemodynamic fluctuations described after acute exercise [2,5,6] were not observed. However, this timing cannot separate sustained from residual short-term effects. Because systemic haemodynamics, autonomic function, and ocular blood flow were not measured, the mechanisms underlying this concordance remain to be determined.

If confirmed, these findings suggest that lower VD and PD in trained athletes may need to be interpreted in the context of training status when OCTA is used in sports medicine [3]. Most previous studies have examined acute exercise responses [8] or short-term interventions such as ocular yoga exercises [18] rather than sustained professional training. Whether OCTA could help distinguish training-associated vascular profiles from early pathological changes, or serve as a biomarker of long-term exercise adaptation, requires validation in prospective studies with repeated OCTA measurements, training-load data, and physiological assessments.

This study has several strengths. The athletes came from one club with a shared coaching staff and training schedule, the groups were similar in age and sex, all imaging was performed on the same device during a defined morning interval, both retinal and choroidal layers were analysed, and multiple testing was controlled with FDR correction alongside effect-size reporting. Several limitations should also be considered. The cross-sectional design precludes causal inference and cannot determine whether the profile developed with training or preceded it. The sample was modest, male-only, and drawn from a single club and centre; it had 80% power only for standardized differences of approximately d = 0.73 or larger, and some outcomes had missing data. Axial length, cumulative training load, exercise intensity, cardiorespiratory fitness (e.g., VO_2_max), systemic haemodynamics, and direct ocular blood-flow measurements were not available. Detailed dietary intake and hydration status were not assessed, and the habitual physical activity of controls was not quantified objectively, so group differences may partly reflect factors other than training. Finally, imaging one day after the last training session cannot distinguish acute from sustained effects.

## 5. Conclusions

Professional handball players had lower OCTA-derived retinal and choroidal vessel and perfusion density than sedentary controls, while FAZ geometry and neuroretinal structural parameters did not differ significantly after multiple-comparison adjustment. Physiological adaptation is one possible explanation, but the cross-sectional data do not establish its mechanism or clinical significance. Longitudinal studies incorporating exercise intensity, cardiorespiratory fitness, dietary assessment, and systemic haemodynamic measurements are needed.

## Figures and Tables

**Table 1:** Demographic and Clinical Characteristics of Professional Handball Players and Sedentary Controls.

Characteristic	Handball Players (*n* = 31)	Sedentary Controls (*n* = 30)	*p* Value
Age, years, mean ± SD	23.42 ± 3.24	23.63 ± 1.52	0.941 ^a^
Age range, years	16–32	21–27	—
IOP, mmHg, mean ± SD	12.3 ± 2.7	12.5 ± 2.0	— ^b^
BCVA (decimal)	1.0	1.0	— ^b^

SD, standard deviation; IOP, intraocular pressure measured by Goldmann applanation tonometry; BCVA, best-corrected visual acuity. ^a^ Mann–Whitney U test (U = 459.5). ^b^ Descriptive only; no between-group test was performed.

**Table 2:** Foveal Avascular Zone Metrics and Structural Optical Coherence Tomography Parameters in Professional Handball Players and Sedentary Controls.

Parameter	*n*	Handball Players	Sedentary Controls	Effect Size	Raw *p*	FDR *p*
FAZ area (mm^2^)	31/30	0.74 ± 0.44 0.50 (0.38–0.99)	0.60 ± 0.35 0.54 (0.41–0.62)	r = −0.072	0.634	0.634
FAZ perimeter (mm)	31/30	5.32 ± 2.16 4.38 (3.65–7.35)	4.48 ± 1.54 4.15 (3.66–4.76)	r = −0.158	0.292	0.317
FAZ circularity	31/30	0.34 ± 0.10 0.32 (0.28–0.37)	0.38 ± 0.08 0.39 (0.33–0.44)	r = 0.306	0.040	0.058
RNFL thickness (µm) ^a^	30/28	98.80 ± 9.46 97.50 (92.62–105.88)	102.34 ± 8.17 102.25 (96.00–107.12)	d = −0.40	0.134	0.174
GCC thickness (µm)	31/18	97.53 ± 9.89 98.00 (93.50–103.00)	100.33 ± 8.11 101.25 (93.75–105.38)	r = 0.188	0.281	0.317

Values are mean ± standard deviation (first line) and median (interquartile range) (second line). *n*, number of analyzed eyes (handball players/controls); FAZ, foveal avascular zone; RNFL, retinal nerve fiber layer; GCC, ganglion cell complex; FDR, Benjamini–Hochberg false discovery rate. Comparisons were performed using the Mann–Whitney U test unless otherwise indicated. ^a^ Independent-samples *t*-test. Negative Cohen’s d and positive rank-biserial r values indicate lower values in handball players. Raw *p* values were adjusted together across the 13 outcomes reported in Table 2 and Table 3.

**Table 3:** Retinal and Choroidal Optical Coherence Tomography Angiography Parameters in Professional Handball Players and Sedentary Controls, Grouped by Vascular Layer.

Parameter	*n*	Handball Players	Sedentary Controls	Effect Size	Raw *p*	FDR *p*
SCP VD (mm/mm^2^)	31/30	5.80 ± 2.37 5.50 (4.56–7.50)	8.71 ± 2.29 9.62 (8.28–10.22)	r = 0.668	<0.001	<0.001
SCP PD (%)	31/30	23.81 ± 10.64 21.30 (15.90–32.25)	35.36 ± 8.25 37.85 (32.00–40.35)	r = 0.626	<0.001	<0.001
DCP VD (mm/mm^2^)	28/29	2.91 ± 2.10 2.00 (1.50–4.38)	5.22 ± 2.49 5.25 (3.25–7.00)	r = 0.552	<0.001	<0.001
DCP PD (%)	29/30	8.22 ± 7.71 5.62 (3.38–9.38)	18.29 ± 11.06 15.25 (9.88–26.22)	r = 0.555	<0.001	<0.001
CC VD (mm/mm^2^)	31/30	6.61 ± 2.68 7.25 (4.44–9.19)	8.44 ± 2.19 9.31 (7.75–9.72)	r = 0.449	0.003	0.004
CC PD (%)	31/30	29.65 ± 15.47 32.00 (16.06–43.12)	42.82 ± 11.45 47.62 (36.97–49.34)	r = 0.574	<0.001	<0.001
Choroid VD (mm/mm^2^)	31/30	5.90 ± 2.65 6.62 (3.38–8.06)	7.90 ± 1.80 8.50 (7.91–9.09)	r = 0.498	0.001	0.002
Choroid PD (%)	31/30	29.86 ± 16.32 33.00 (15.25–44.60)	42.16 ± 9.94 46.95 (39.05–48.20)	r = 0.467	0.002	0.003

Values are mean ± standard deviation (first line) and median (interquartile range) (second line) of the inner- and outer-ring ETDRS measurements. *n*, number of analyzed eyes (handball players/controls); SCP, superficial capillary plexus; DCP, deep capillary plexus; CC, choriocapillaris; VD, vessel density; PD, perfusion density; ETDRS, Early Treatment Diabetic Retinopathy Study; FDR, Benjamini–Hochberg false discovery rate. All comparisons were performed using the Mann–Whitney U test. Positive rank-biserial r values indicate lower values in handball players. Raw *p* values were adjusted together across the 13 outcomes reported in Table 2 and Table 3.

## Data Availability

The data presented in this study are available from the corresponding author upon reasonable request, due to institutional data protection restrictions and the absence of participant consent for public data deposition.
